# Community-based randomised controlled trial evaluating falls and osteoporosis risk management strategies

**DOI:** 10.1186/1745-6215-9-62

**Published:** 2008-11-04

**Authors:** PM Ciaschini, SE Straus, LR Dolovich, RA Goeree, KM Leung, CR Woods, GM Zimmerman, SR Majumdar, S Spadafora, LA Fera, HN Lee

**Affiliations:** 1 Algoma District Medical Group, Sault Ste. Marie, Canada; 2 Department of Medicine, University of Calgary, Calgary, Canada; 3 Department of Medicine, University of Toronto, Toronto, Canada; 4 Department of Family Medicine, McMaster University, Hamilton, Canada; 5 Centre for Evaluation of Medicines, St. Joseph's Healthcare, Hamilton, Canada; 6 Program for Assessment of Technology in Health (PATH) Research Institute, St. Joseph's Hospital, Hamilton, Canada; 7 Department of Clinical Epidemiology and Biostatistics, McMaster University, Hamilton, Canada; 8 Department of Physical Therapy, Group Health Centre, Sault Ste. Marie, Canada; 9 Algoma Public Health, Sault Ste. Marie, Canada; 10 Department of Biology, Lake Superior State University, Sault Ste. Marie, USA; 11 Department of Medicine, University of Alberta, Edmonton, Canada; 12 Clinical Research Department, Group Health Centre, Sault Ste. Marie, Canada; 13 Department of Epidemiology and Biostatistics, Schulich School of Medicine and Dentistry, the University of Western Ontario, London, Canada

## Abstract

**Background:**

Osteoporosis-related fractures are a significant public health concern. Interventions that increase detection and treatment of osteoporosis, as well as prevention of fractures and falls, are substantially underutilized. This paper outlines the protocol for a pragmatic randomised trial of a multifaceted community-based care program aimed at optimizing the evidence-based management of falls and fractures in patients at risk.

**Design:**

6-month randomised controlled study.

**Methods:**

This population-based study was completed in the Algoma District of Ontario, Canada a geographically vast area with Sault Ste Marie (population 78 000) as its main city. Eligible patients were allocated to an immediate intervention protocol (IP) group, or a delayed intervention protocol (DP) group. The DP group received usual care for 6 months and then was crossed over to receive the interventions. Components of the intervention were directed at the physicians and their patients and included patient-specific recommendations for osteoporosis therapy as outlined by the clinical practice guidelines developed by Osteoporosis Canada, and falls risk assessment and treatment. Two primary outcomes were measured including implementation of appropriate osteoporosis and falls risk management. Secondary outcomes included quality of life and the number of falls, fractures, and hospital admissions over a twelve-month period. The patient is the unit of allocation and analysis. Analyses will be performed on an intention to treat basis.

**Discussion:**

This paper outlines the protocol for a pragmatic randomised trial of a multi-faceted, community-based intervention to optimize the implementation of evidence based management for patients at risk for falls and osteoporosis.

**Trial Registration:**

This trial has been registered with clinicaltrials.gov (ID: NCT00465387)

## Background

Osteoporosis, a chronic condition characterized by decreased bone mass and increased risk of fractures, is a significant public health concern. It affects over 200 million people worldwide[1], an estimated 10 million people in the US[2], 4 million people in the UK[3] and 1.4 million people in Canada[2,4]. Fragility fractures are the clinical consequence of osteoporosis. While vertebral fractures can cause back pain, loss of height and disability, hip fractures have a more significant impact on quality of life leading to loss of function, and admission to long-term care. [5-9] It is estimated that 1 in 5 people who suffer a hip fracture will die during the first year and less than one third gain their pre-fracture level of function. [2] Moreover, the economic impact of this condition is considerable, with the total acute care cost of osteoporosis estimated to be over $1.3 billion per year in Canada[9], $20 billion in the US[10] and over €30 billion in Europe. [11,12] Given that the proportion of people aged 65 and older is increasing, this will likely lead to an even more significant burden of disease. [4,12]

Osteoporosis increases the risk of fractures in elderly patients who fall. [4] Falls are the leading cause of accidental death among people aged 65 years of age and older, and also account for significant morbidity, including fracture, impaired mobility, decreased quality of life due to fear of falling, depression, admission to long-term care facilities, and death. [13-18] Estimates vary, but approximately 20% of falls among the elderly result in serious injury requiring medical attention, and 2% to 10% of these falls result in fractures. [13,14,18-21] Because of the morbidity and mortality associated with falls, they result in marked costs for the health care system and are a major health concern. [22]

For the prevention of fractures, a substantial body of evidence supports detection and treatment of osteoporosis in high-risk patients, including appropriate bone mineral density testing and therapy with calcium, vitamin D, and drugs that decrease bone resorption or increase bone formation. [23] For example, bisphosphonates reduce the risk of future osteoporosis-related fracture by 40 to 60%, with fracture reduction apparent within a year of starting treatment. [23] Similarly, research suggests that interventions targeting both intrinsic and environmental risk factors reduce the risk of falls in older people. [24]

Despite the incorporation of this evidence from randomized trials into clinical practice guidelines, these interventions are considerably underutilized. [25-29] According to a recent systematic review, the proportion of individuals with a fragility fracture who received a diagnostic test for osteoporosis or a diagnosis from a physician ranged from 1.7% to 50%. [27] And, it is unclear how many patients who experience a fracture are assessed for risk of falls.

These care gaps highlight the finding that additional effort is needed to ensure that appropriate knowledge translation is achieved to optimize management of patients at risk for falls and fracture. This study was designed to help fill this knowledge to practice gap. The primary objective of this study was to determine if a multicomponent, complex, community-based, integrated care strategy could optimize the evidence-based and guideline-concordant management of people at high risk for falls and fractures. This study was developed by a community partnership because of a locally identified need to address these evidence to practice gaps. Given the significant disease burden of falls and fractures in the local community, the partnership decided to implement and evaluate a multi-faceted intervention to enhance care.

## Methods

Between March 2003 and January 2006, we conducted a randomized trial of a multifaceted community based intervention to optimize care of patients at risk for falls and fractures. Two parallel trials were conducted in this group of patients. We did not conduct a factorial trial because we had no reason to believe that osteoporosis treatment and falls assessment would not be managed independently. Previous studies have shown this to be true. [30,31] An economics evaluation was carried out in parallel to this randomized controlled trial to evaluate the costs and consequences as well as cost-effectiveness of applying the intervention.

### Setting and Study Population

This study was completed in the Algoma District of Ontario, Canada, a geographically vast area with Sault Ste Marie (population 78 000) as its main city. The study represented a partnership among consumers, providers and other stakeholders interested in reducing falls and fractures in the community. It was conducted by the Group Health Centre (GHC), a multidisciplinary, multi-specialty, not-for-profit health service community centre, in partnership with Sault Area Hospital (SAH), a facility with 250 active beds. When the study was initiated, the Group Health Centre had 44 000 registered patients. Distinguishing features of the GHC are multiple types of health care professionals integrated within a health care delivery system, capitation funding and an electronic medical record system (EMR). The study was also completed in partnership with the Algoma Community Care Access Centre; the Algoma Health Unit; and the Slips, Trips and Falls Committee of Sault Ste Marie Safe Communities Partnership, a community-based falls prevention coalition.

Patients were eligible for inclusion in the study if they were community-dwelling, aged 55 years or older, able to give informed consent, and were identified to be at risk for a future fracture according to one of the following criteria:

1. attended the hospital Fracture Clinic for a non-pathological fracture of the vertebrae, hip or wrist or BMD in the past year with a T-score of ≤ -2.0;

2. attended the hospital Emergency Department with a fall and found to be at high risk for falls as defined by a Timed Up and Go[32] result of greater than 14 seconds; or,

3. were self-referred or referred by a health care provider because of perceived high risk of fracture, and identified as a high risk for falls defined by a Timed Up and Go result of greater than 14 seconds.

Patients were excluded if they were already receiving appropriate therapy for osteoporosis as outlined in the Osteoporosis Canada guidelines. [23]

### Interventions

#### Osteoporosis Risk Assessment and Management

This intervention was multifaceted and consisted of providing evidence-based management strategies for osteoporosis to both the patients and their primary care providers. Following randomization, a bone mineral density (BMD) test was facilitated for participants if it had not been done within the past year, and the results were sent to the primary care physician along with relevant prescribing information based on the Osteoporosis Canada guidelines. [13] A complete list of the patient's medications was compiled from two sources and provided to physicians: (1) the patient's pharmacy records; and (2) home visits conducted by the study nurses. Medications associated with increased risk of fracture were identified, and physicians were asked to assess this list of flagged medications (Appendix).

Patients received personalized counseling from the research nurse about osteoporosis, including a written summary of the proposed management plan. They also received educational materials on calcium and vitamin D, risk factors for osteoporosis, and their BMD results. For those few individuals without a primary care physician, the same materials were provided but patients were encouraged to visit a walk-in clinic or the emergency department.

### Falls assessment and management

The intervention was multifaceted and consisted of providing patient-specific evidence-based recommendations targeted to reduce falls risk to both the patients and their primary care providers. A research nurse assessed participants in their home and completed the Berg Balance Scale[33], the InterRAI Screener[34,35], a medication review and an assessment for orthostatic hypotension. The InterRai screener is used to assess the elderly individual to identify those who merit further assessment in order to prevent or stabilise early functional or health decline. Medications associated with increased risk of falls were identified through review of pharmacy records and home visits and primary care providers were asked to assess this list of flagged medications (Appendix).

The criteria for appropriate referral for physiotherapy and occupational therapy services were based on the results of the InterRAI Screener and the Berg Balance Score. Physiotherapy interventions were tailored to each patient and included strengthening exercises, gait and balance training, and referral to activities such as T'ai Chi classes. Occupational therapy interventions were also tailored to each patient and included home environmental assessment and cognitive testing. Details on the interventions provided by the rehabilitation therapists will be described in additional detail in the results paper for this study. All therapists completed standard reporting forms indicating their recommended interventions and barriers to patient compliance with these recommendations.

Patients received personalized counseling from the research nurse about fall prevention including a written summary of the suggested management plan. They also received educational materials including a checklist for falls prevention.

### Allocation and Blinding

Eligible patients were randomized using a computer generated randomization scheme under supervision of the study biostatistician, into an immediate intervention protocol (IP) group or to a delayed intervention protocol (DP) group. All members of the DP group received usual health care during the first 6 months of the main study and then were able to receive the intervention after this period. Patients, physicians and outcomes assessors could not be blinded to the fact that patients were part of a falls and fracture prevention study. All patients were followed-up at 6 and 12 months after the completion of the initial assessment.

### Outcomes and Data Collection

The primary outcome for the osteoporosis study was appropriate osteoporosis management based on the 2002 clinical practice guidelines for osteoporosis in Canada. [23] This was the most current evidence-based guideline available for use at the time of study onset and was generally consistent with US and UK guidelines. The primary outcome for the falls study was the implementation of appropriate falls risk assessment at 6 months. Measurements of outcomes were obtained through patient records (obtained through the Electronic Medical Record), therapists' reports, and pharmacy data. A standardized chart review of the Electronic Medical Record was the primary source of data for both groups.

Secondary outcomes included the number of falls and fractures as recorded in monthly patient diaries. Follow-up telephone calls every 3 months were used to obtain this data and completed patient diaries were mailed to the investigators at study end. Quality of life was measured using the OPTQoL[36], a disease-specific quality of life questionnaire. After the main study was completed at 6-months, and the DP group crossed-over to receive the intervention, all patients were followed up for a further 6 months.

### Economics substudy

The costs that will be included are: nursing time, administrative time (e.g. booking appointments), home visit travel, costs of health care professional continuing education, costs of community education, and the costs of program materials. Interviews with program staff and cost information from the GHC administration will be used to calculate program costs. Research project evaluation costs will be excluded from program costs. Costs to the health care system as well as costs to the patient will be collected from the GHC electronic medical record, the Sault Area Hospital medical record, or via patient interview. The intervention program costs and health resource utilization were collected at 6 months post-enrollment, 1 year post-enrollment and 2 years post-enrollment. Analyses will be done from the perspectives of the public payer and the patient. Effectiveness and cost information will be applied to a decision analytic model that has been specifically designed to determine the cost effectiveness of various treatments for osteoporosis. To assess the long term cost-effectiveness of the program for both men and women of various ages, separate long term projection models for both men and women by age would be required. The only model that is currently available is a model based on post-menopausal osteoporosis. [37,38] Therefore, it is in this patient population that the long term cost-effectiveness will be estimated based on the intermediate outcomes from this study.

### Qualitative substudy

A sample of program staff, patients and physicians will be interviewed about their experiences in the study and to explore issues around costs, adherence to the intervention and whether the material on osteoporosis and falls prevention was discussed by patients and physicians. Individual, semi-structured interviews will be facilitated by an experienced interviewer. Purposive sampling will be used to identify patients who did and did not receive appropriate therapy. Their primary care physicians will also be invited to participate in individual interviews. Domains of enquiry will focus on knowledge of osteoporosis and falls prevention information and perceptions of use of this evidence in decision making and clinical care. Barriers and facilitators to implementation will also be explored. Interviews will be audiotaped and the tapes will be transcribed. Any unique participant identifiers will be removed from the transcripts. Two investigators will independently analysis the transcripts using content analysis to identify themes. NVivo will be used to analyse the data. Investigators will use the constant comparative method to challenge the development of their themes and will use memos to track the development of their themes and analytical strategy.

### Sample size and Analysis

Local pilot data suggested that 40% of people would receive appropriate osteoporosis medications at 6 months. Similarly, it was anticipated that 40% of people would receive appropriate falls assessment at 6 months. We determined a minimal clinically important difference of 20% and with a two-tailed alpha of 0.05, power of 0.80, the patient as the unit of allocation and analysis, and assuming a loss to follow-up of 10%, a total sample size of 200 patients was needed.

Descriptive statistics will be calculated for all variables of interest. Continuous measures will be summarized using means and standard deviations and categorical measures will be summarized using percentages. The principal outcome measure will be assessed using a two sample two-sided test of proportions and will be performed on an intention to treat basis.

Ethics approval was received from the Joint Group Health Centre/Sault Area Hospital Research and Ethics Board. This trial has been registered with clinicaltrials.gov (ID: NCT00465387)

## Discussion

It is critical that the health care community address the deficiencies that exist with respect to knowledge translation and management of osteoporosis given the significant burden of disease associated with falls and fractures. Implementing evidence-based strategies with timely follow-up and treatment is especially important in acute-care settings (e.g. fracture clinics and hospitals), since the individuals who present in these settings are often at highest risk for future sequelae. [39,40] The study will be able to evaluate the clinical and cost impact of a multidisciplinary intervention program for falls risk and osteoporosis management on appropriate assessment and treatment compared to usual care in a northern community. Its design is pragmatic and is focused on ensuring that a local program is evaluated before a decision is made to implement it long-term. The combination of clinical and cost evidence generated is essential information for health care decision makers assessing the long term viability of the program.

Limitations of this trial include that randomization will occur at the level of the patient rather than at the practice level and a short follow up time of 6 to 12 months. Because of the small number of primary care practices in this location, we are unable to randomize at the practice level. Given that that it is extremely difficult to change behaviour[41], and that there are significant time constraints to the primary care visits in this region due to physician shortages, we anticipate that this will not have a significant impact on the study outcomes.

The findings from this trial will provide information of interest to many stakeholders including patients, health care managers, public health officials, policy makers, and health care professionals amongst others. To our knowledge, it represents the first Canadian trial attempting to bridge the evidence-to-practice gap around osteoporosis and falls prevention. Moreover, it includes a successful partnership amongst relevant community stakeholder groups which is required to ensure appropriate implementation of a multi-faceted intervention and to facilitate sustainability of effective interventions.

## Competing interests

The authors declare that none of them have received honoraria, reimbursement or fees from any pharmaceutical companies. However, several pharmaceutical companies provided funding for completion of this research project including Merck Frosst Canada Ltd., Sanofi-Aventis Pharma Inc., Proctor & Gamble Pharmaceuticals Canada Inc., and Eli Lilly Canada Inc. Financial support for the completion of this project was also received from the Greenshield Foundation. Equipment (e.g. office space, computers, telephones) was contributed in-kind by the Group Health Centre, Algoma Public Health, Sault Area Hospital, Algoma Community Care Access Centre, and the Slips, Trips and Falls Committee of Sault Ste. Marie Safe Communities Partnership, all located in Sault Ste. Marie, Ontario.

## Authors' contributions

PC (Primary investigator) was responsible for interpretation of the data, implementation of the study and was involved with drafting and review of the manuscript. She assumed this role after the death of the principal investigator, Dr. H. Lee. SES (co-investigator) was responsible for interpretation of data, drafting of the manuscript and assisted with the implementation of the study after the death of the PI. LRD (co-investigator) was involved with study design, interpretation of data, and drafting and review of the manuscript. RGG (co-investigator) was involved with the study design and analysis of the results. KML (co-investigator) was involved with study design, implementation of the study and review of the manuscript. CRW (co-investigator) was involved with study design, implementation of the study and review of the manuscript. GMZ (biostatistician) was responsible for study design, randomization, analysis and interpretation of the data, and has been directly involved with review of the manuscript. SRM (co-investigator) was involved with study design, interpretation of the results, and drafting and reviewing of the manuscript. SS (collaborator) was involved with review of the manuscript. LAF (co-investigator) was involved with analysis and interpretation of the data, and drafting of the manuscript. HNL (primary investigator) was the primary investigator and responsible for the study design and its implementation.

## Appendix: risk medications 

Medications associated with an increased risk of falls:

1. *Benzodiazepines*: alprazolam, midazolam, clonazepam, delavirdine, diazepam, halazepam, lorazepam, chlordiazepoxide, oxazepam, prazepam, quazepam, temazepam, triazolam.

2. *Tricyclic Antidepressants*: amitriptyline (Elavil), amoxaine (Asendin), clomipramine (Anafranil) desipramine (Norpramin), dothiepin (Prothiaden), doxepin (Sinequan), imipramine (Tofranil), nortriptyline (Aventyl), trimipramine (Surmontil), lofepramine (Gamanil).

3. *Direct-Acting Vasodilators*: doxazosin (Cardura), hytrin (Terazosin)

Medications associated with an increased risk of osteoporosis:

1. *Glucocorticoids*: Cortef, cortisone, dexamethasone, hydrocortisone, prednisone, betamethasone, fludrocortisone, methylprednisone, prednisolone, tramcinolone.

2. *Inhaled Corticosteroids*: beclomethasone, budesonide, fluticasone, flunisolide, tramcinolone.
